# Safety and Efficacy of Metabolic Surgery in Patients with Type 2 Diabetes in the Middle East and North Africa Region: An Analysis of Primary Roux-en-Y Gastric Bypass and Sleeve Gastrectomy Outcomes

**DOI:** 10.3390/jcm12155077

**Published:** 2023-08-02

**Authors:** Sami Fares, Juan S. Barajas-Gamboa, Gabriel Díaz del Gobbo, Michael Klingler, Juan Pablo Pantoja, Carlos Abril, Javed Raza, Alfredo D. Guerron, Ricard Corcelles, Matthew Allemang, John Rodriguez, Matthew Kroh

**Affiliations:** 1 Department of Surgery, Case Western Reserve University School of Medicine, Main Campus, Cleveland, OH 44106, USA; 2 Department of General Surgery, Cleveland Clinic Abu Dhabi, Abu Dhabi P.O. Box 112412, United Arab Emirates; barajaj@clevelandclinicabudhabi.ae (J.S.B.-G.); delgobg@clevelandclinicabudhabi.ae (G.D.d.G.); pantojj@clevelandclinicabudhabi.ae (J.P.P.); abrilc@clevelandclinicabudhabi.ae (C.A.); rodrigj2@clevelandclinicabudhabi.ae (J.R.); 3 Department of General Surgery, Cleveland Clinic, Cleveland, OH 44195, USA; klinglm@ccf.org (M.K.); allemam@ccf.org (M.A.); 4 Cleveland Clinic Lerner College of Medicine, Case Western Reserve University, Cleveland, OH 44106, USA

**Keywords:** metabolic surgery, sleeve gastrectomy, Roux-en-Y gastric bypass, type 2 diabetes

## Abstract

Introduction: Type 2 diabetes (T2D) is a chronic medical condition that results in significant health implications and reduced life expectancy. The International Diabetes Federation (IDF) estimated that in 2021, 51.8% of all deaths of people under 60 years old in the Middle East and North Africa (MENA) region were related to diabetes. Bariatric surgery has been demonstrated to be a safe and effective treatment for T2D in different populations worldwide, though few specific data exist on outcomes of procedures in the MENA region. The aim of this study was to compare the safety and postoperative outcomes between patients with and without T2D undergoing primary bariatric surgery at a tertiary referral academic medical center in the United Arab Emirates. Methods: All patients who underwent primary metabolic surgery between September 2015 and July 2020 were retrospectively reviewed from a prospective database. Group 1 included patients with T2D, and Group 2 included patients without T2D. Patients undergoing revisional or correctional operations were excluded. The procedure performed was based on surgeon discretion in discussion with a multidisciplinary team and the patient. Demographics as well as perioperative and postoperative results were examined. Results: Our study included 542 patients, 160 (29.5%) with T2D and 382 (70.5%) with non-T2D. Mean age was 44.5 years (range 16–70) in the T2D group and 33.3 years (range 15–63) in the non-T2D group; median BMI was 41.8 ± 7.3 and 43.2 ± 7.2, respectively. The T2D group was 37.5% male and 62.5% female, and the non-T2D group was 38.7% male and 61.3% female. There were no significant differences in comorbidities. In the T2D group, 45.6% of patients underwent Roux-en-Y gastric bypass and 54.4% sleeve gastrectomy. In the non-TD2 group, 42.7% of patients received Roux-en-Y gastric bypass and 57.3% sleeve gastrectomy. There were no statistically significant differences in postoperative ED visits (21.8% vs. 24.3%, *p =* 0.21), minor complications within 30 days (4.3% vs. 5.2%, *p =* 0.67), readmission rates (5.6% vs. 4.9%, *p =* 0.77), re-operation rates (3.7% vs. 1.5%, *p =* 0.11), median hospital stay (2.0 days vs. 3.0, *p =* 0.05), or complications after 30 days (6.2% vs. 11.2%, *p =* 0.07). There were no deaths either group. Conclusions: In this cohort of patients from the MENA region, bariatric surgery in T2D patients is safe and effective, with perioperative outcomes comparable to those of non-T2D patients. To the best of our knowledge, our postoperative findings, which are the first report in the MENA region, are consistent with studies published in North America and Europe.

## 1. Introduction

Obesity has emerged as a significant public health challenge in the Middle East, particularly in the United Arab Emirates (UAE). The region has witnessed a substantial rise in obesity rates over the past few decades, leading to numerous health consequences and placing a significant burden on the healthcare system [1]. Employing scientific language, examining relevant statistics, and highlighting the impact on healthcare, this document aims to provide an overview of obesity in the Middle East, with a specific focus on the United Arab Emirates [1,2].

Statistics reveal alarming rates regarding obesity in the UAE. Recent studies indicate that the prevalence of obesity in the country has reached critical levels, with approximately 37% of the population classified as obese. This places the UAE among the countries with the highest rates of obesity worldwide. Furthermore, there has been a notable increase in childhood obesity, with estimates suggesting that around 15% of children in the UAE are obese. These rates are concerning due to the long-term health implications associated with obesity, both in childhood and adulthood [2].

The impact of obesity on the healthcare system is multifaceted and far-reaching. Obesity is closely associated with a wide range of chronic conditions and comorbidities, including type 2 diabetes (T2D), cardiovascular diseases, hypertension, certain cancers, and musculoskeletal disorders. These obesity-related diseases impose a substantial burden on the healthcare system, requiring extensive resources, specialized care, and long-term management. The costs associated with the treatment and management of obesity-related conditions, including medications, hospitalizations, surgeries, and rehabilitation, contribute to the economic strain on the healthcare system [3,4].

In addition to the direct healthcare costs, obesity also has indirect consequences that affect productivity, quality of life, and societal well-being. Individuals with obesity often experience reduced work productivity, increased sick leave, and higher rates of disability compared to their non-obese counterparts. Moreover, obesity-related conditions can lead to premature mortality, reducing the overall life expectancy and further impacting the workforce and the economy. The indirect costs, such as lost productivity and increased social welfare support, place an additional burden on the healthcare system and society as a whole [5].

In recent times, there has been increased focus on the correlation between obesity and T2D. Extensive research conducted in the scientific community has shed light on the robust and complex relationship between these two conditions. Individuals with higher body mass index (BMI) face a significantly increased risk of developing T2D, with obesity being associated with up to a sevenfold greater risk compared to those with a normal BMI.^6^ This link between obesity and T2D has profound implications on both individual health and societal burdens. T2D is known to contribute to significant morbidity, leading to complications such as cardiovascular disease, neuropathy, retinopathy, and renal dysfunction. Additionally, the management and treatment of T2D impose a substantial economic burden on healthcare systems [6].

In the Middle East and North Africa (MENA) region, the prevalence of T2D has reached alarming proportions. According to 2021 estimates by the International Diabetes Federation (IDF), the overall prevalence of T2D in the MENA region is 13.7%, with a particularly high prevalence of 19.3% among individuals native to the region [7]. These statistics underscore the urgent need for effective management strategies to mitigate the impact of T2D in the region. Furthermore, the IDF reports that T2D-related diseases account for 24.5% of deaths among individuals of working age (15–64) in the MENA region, making it the region with the highest rate of T2D-related deaths in this population globally [8]. These figures highlight the critical importance of addressing T2D as a public health priority in the MENA region.

Over the past few years, metabolic surgery has gained recognition as a widely accepted and successful intervention in the global management of T2D across diverse populations. This surgical approach encompasses procedures such as gastric bypass, sleeve gastrectomy, and duodenal switch and has demonstrated remarkable long-term outcomes for both diabetic and non-diabetic patients [9]. Numerous randomized control trials have consistently shown the superiority of metabolic surgery over conventional medical therapy in obese patients with T2D. These studies have revealed greater weight loss, improved glycemic control, increased insulin sensitivity, and higher rates of T2D remission compared to non-surgical interventions [10,11].

One notable study that highlights the transformative potential of metabolic surgery is a retrospective multicenter study conducted by the Cleveland Clinic in 2011. This study involved a large cohort of 135,000 patients with T2D, of whom an impressive 87% achieved improved glycemic control following metabolic surgery. Moreover, 78% of these patients were able to discontinue their antidiabetic medications entirely, illustrating the profound impact of metabolic surgery on T2D management [9].

Despite the documented benefits of metabolic surgery, concerns regarding its safety in patients with T2D persist. The potential risk of perioperative complications necessitates careful consideration and patient selection. A retrospective study conducted in 2019 examined the outcomes of 1525 patients who underwent metabolic surgery and revealed that diabetic patients faced a higher risk of postoperative mortality (2.5%) as well as complications such as delayed extubation, circulatory dysfunction, incision infections, pulmonary infections, acute myocardial infarction, and acute kidney failure when compared to non-diabetic patients. These findings were largely attributed to factors such as intraoperative hyperglycemia and inadequate preoperative disease management [12].

Furthermore, a cohort study published in the Annals of Surgery in 2015 compared the outcomes of 3200 non-T2D patients undergoing general surgery with 7800 T2D patients undergoing general surgery. The study revealed a 4% increased risk of complications in the T2D group, which was highly correlated with perioperative hyperglycemia. Interestingly, the non-T2D cohort demonstrated similar or even greater risks of adverse events in the presence of perioperative hyperglycemia, underscoring the significance of glycemic control in surgical outcomes [13].

Despite the wealth of evidence supporting the safety and efficacy of metabolic surgery in diverse populations, there remains a notable gap in the literature regarding the outcomes of metabolic surgery specifically within the MENA region, particularly among patients with T2D. This knowledge gap poses significant challenges for healthcare providers and policymakers in the region as they strive to implement evidence-based strategies for managing T2D. As such, the aim of this study was to compare the safety and postoperative outcomes between patients with and without T2D undergoing primary bariatric surgery at a tertiary referral academic medical center in the United Arab Emirates.

## 2. Methods

### 2.1. Study Design and Ethical Approvals

This retrospective review of a prospective database was conducted between September 2015 and July 2019. Specific data on indications for primary metabolic surgery, including sleeve gastrectomy (SG) and Roux-en-Y gastric bypass (RYGB), perioperative outcomes, and postoperative outcomes during follow-up, were collected and analyzed. The preparation of this manuscript followed the STROBE guidelines. This study was approved by the Research Ethics Committee (REC) at our institution under the internal number A-2017-029.

### 2.2. Objectives

The aim of this study was to compare the safety and postoperative outcomes between patients with and without T2D undergoing primary bariatric surgery at a tertiary referral academic medical center in the United Arab Emirates.

### 2.3. Population

All patients were divided into two groups: patients with T2D and patients without T2D. The definition of T2D was based on the American Diabetes Association (ADA) criteria, which includes a fasting blood glucose greater than or equal to 126 mg/dL, an HbA1c greater than 6.5%, or an oral glucose tolerance test result greater than or equal to 200 mg/dL.

### 2.4. Inclusion and Exclusion Criteria

Patients older than 18 years and younger than 70 years, with a BMI greater than or equal to 30 kg/m^2^, undergoing primary SG and RYGB during the study period were included. Patients undergoing revision of previous SG and RYGB, as well as those younger than 18 years and older than 70 years, were excluded.

### 2.5. Preoperative Management

The preoperative work-up included evaluation by our multidisciplinary team for patients undergoing primary metabolic surgery. Preoperative investigations included esophagogastroduodenoscopy (EGD), contrast-enhanced upper gastrointestinal series, and blood chemistry panels. Abdominal computerized tomography scans and/or abdominal ultrasounds were obtained at the discretion of the treating physicians.

### 2.6. Surgical Techniques

#### 2.6.1. SG

The patient was brought to the operating room and identified by name, medical record number, and date of birth. A sign-in huddle was performed by the anesthesia and surgical teams. The patient was placed in a supine position on the operating table. The abdomen was prepped and draped in the usual sterile fashion. A surgical timeout was performed. The peritoneal cavity was entered with a 5 mm optical trocar in the left upper quadrant. Pneumoperitoneum was established. Bilateral transversus abdominis plane (TAP) blocks were performed. Trocars were gently placed in a U shape.

The Nathanson liver retractor was inserted under direct observation. We began dissection by taking down the phrenoesophageal fat pad and exposing the angle of His. We mobilized the greater curve by dividing the gastrocolic ligament. We extended it distally to about 5 cm from the pylorus. Posterior short gastrics were divided to completely mobilize the fundus. We inserted a 40 Fr Bougie and constructed the sleeve using sequential applications of the gastrointestinal anastomosis (GIA) stapler with staple reinforcement. Hemostasis was excellent. The specimen was removed, and the port was closed with a figure-of-8 #0 Vicryl stitch (Johnson & Johnson, Somerville, NJ, USA). The endoscope was then inserted through the mouth under direct observation and advanced to the duodenum. The sleeve was clear and exhibited no evidence of leakage. The retractor was removed and the pneumoperitoneum evacuated. The skin was closed with 4-0 Monocryl (Johnson & Johnson, Cornelia, GA, USA). The patient tolerated the procedure well and was taken to the post-anesthesia care unit (PACU) in a stable condition.

#### 2.6.2. RYGB

The patient was brought to the operating room and identified by name, medical record number, and date of birth. A sign-in huddle was performed by the anesthesia and surgical teams. The patient was placed in a supine position on the operating table. The abdomen was prepped and draped in the usual sterile fashion. A surgical timeout was performed. The peritoneal cavity was entered with a 5mm optical trocar in the left upper quadrant. Pneumoperitoneum was established. Bilateral TAP blocks were performed. Trocars were gently placed in a U shape. The Nathanson liver retractor was inserted under direct observation.

We then identified the left gastric pedicle. Just distal to this, the descending branch was transected with a reinforced purple load. Sequential applications of the endo-GIA stitch (Medtronic, Minneapolis, MN, USA) were used to construct a small gastric pouch. We then identified the ligament of Treitz, and, 100 cm distal to this, we transected the bowel with a single application of the GIA stapler using a tan load. Additional mesentery was divided with the US dissector. We measured a 100 cm Roux limb and constructed a side-to-side jejunojejunostomy with a single application of the 60 mm GIA stapler using a tan load. The enterotomy was closed with running 2-0 Vicryl (Johnson & Johnson, Somerville, NJ, USA).

The mesenteric defect was closed with 2-0 Ethibond (Johnson & Johnson, Cornelia, GA, USA). An omental split performed. The gastrojejunostomy was then constructed with a linear stapler. The Petersen space was closed with running 2-0 Ethibond. All 12 mm trocars were closed with #0 Vicryl using the Carter-Thomason method. The bowel was occluded, and the front viewing endoscope was inserted through the mouth under direct observation and advanced to the jejunum. Anastomosis was evident, with no evidence of intraluminal bleeding or leakage. The retractor was removed and pneumoperitoneum evacuated. The skin was closed with 4-0 Monocryl. Patient tolerated the procedure well and was taken to the PACU in a stable condition.

#### 2.6.3. Postoperative Management

Postoperatively, patients were admitted to the surgical ward according to a standardized recovery protocol consisting of early ambulation, multimodal narcotic-sparing analgesia, and initiation of a clear liquid diet the day following surgery. After monitoring for perioperative complications, patients were discharged home when tolerating adequate oral intake. Patients followed up with evaluations in our outpatient clinic with members of our multidisciplinary team at intervals of one week, one month, and then every six months postoperatively.

#### 2.6.4. Data Collection and Statistical Analysis

Data were collected prospectively in an institutional database approved by the REC and retrospectively reviewed, including but not limited by patient demographics, indications for procedure, operative times, and postoperative recovery parameters such as length of hospitalization, reoperation, operative complications, and mortality.

Descriptive statistics were computed for all variables. For continuous variables, these include mean, median, standard deviations and percentiles, and categorical variables were summarized using frequencies and percentages. Comparisons were completed using parametric or non-parametric methods where appropriate, and a significance level of *p* < 0.05 was used to determine statistical significance. The comparison test included “independent sample *t* tests” when examining continuous variables, and “Fisher’s exact test” when examining dichotomous variables. All analyses were carried out using R (version 2.13 or higher, The R Foundation for Statistical Computing, Vienna, Austria).

### 2.7. Study Limitations

This retrospective study, despite utilizing prospectively collected patient data, has inherent limitations that need to be considered. The authors’ selection of cases introduces the potential for selection bias due to a non-randomized process, which may lead to preferential inclusion or exclusion of certain patients and potentially distort the results. Moreover, relying on available medical records poses a risk of incomplete or missing data, compromising the accuracy and validity of the findings. Therefore, it is essential to take these limitations into account when interpreting the study’s outcomes and generalizing the results to broader populations or settings.

### 2.8. Study Strengths

This is the first study conducted in the MENA region comparing the safety and postoperative outcomes between patients with and without T2D undergoing primary bariatric surgery.

## 3. Results

There was a total of 542 patients included in the study, with 160 patients (29.5%) in the T2D cohort and 382 patients (70.5%) in the non-T2D cohort. The baseline characteristics within the cohorts were comparable, ensuring a balanced representation of patients (Table 1). The mean age of patients in the T2D cohort was 44.5 years, whereas in the non-T2D cohort it was 33.1 years. The initial median BMI for the T2D cohort was 41.8, while for the non-T2D cohort it was 43.2 8 kg/m^2^. The gender distribution in the T2D cohort was 37.5% males and 62.5% females, while in the non-T2D cohort it was 38.7% males and 61.3% females. The initial median HbA1c for the T2D cohort was 7.2, while for the non-T2D cohort it was 5.4. Importantly, both cohorts exhibited similar comorbidity profiles.

Examining comorbid conditions, it was found that among all patients, 95 individuals (59.3%) in the T2D cohort had hypertension, compared to 91 patients (23.8%) in the non-T2D cohort (*p* = 0.00001). Moreover, in the T2D and non-T2D cohorts, 71.2% and 35.6%, respectively, had hyperlipidemia (*p* = 0.00001).

With regard to the surgical procedures, 45.7% of patients in the T2D group underwent RYGB, while 54.3% underwent SG. In the non-T2D group, 42.7% received RYGB, and 57.3% underwent SG (Table 2). Notably, no statistically significant differences were observed in intraoperative findings. The average duration of surgery was 117 min in the T2D cohort and 114 min in the non-T2D cohort, showing a non-significant trend (*p* = 0.057218).

Analysis of perioperative outcomes revealed no statistically significant differences between the two cohorts (Table 3). The mean length of hospital stay did not differ significantly, with T2D patients staying for an average of 3 days compared to 2 days for non-T2D patients (*p* = 0.052873). Early minor complications were observed in 4.3% of T2D patients and 5.2% of non-T2D patients, with no significant difference between the groups (*p* = 0.67448). Similarly, early major complications occurred in 4.3% of T2D patients and 3.9% of non-T2D patients, demonstrating no significant difference in complication rates (*p* = 0.81034). Readmission rates within the early postoperative period were 5.6% for T2D patients and 4.9% for non-T2D patients, without a statistically significant distinction (*p* = 0.75656).

Furthermore, reoperation rates were 3.7% for T2D patients and 1.5% for non-T2D patients, with no significant difference observed (*p* = 0.11642). Interestingly, there was no statistically significant difference in the mean BMI at 12 months of follow-up between T2D and non-T2D cohorts, indicating similar weight loss outcomes (30.2 kg/m^2^ for T2D vs. 29.1 kg/m^2^ for non-T2D, *p* = 0.777767). However, a significant difference was found in the mean HbA1c levels at 12 months of follow-up, with T2D patients showing an average HbA1c of 5.7 compared to 5.2 in non-T2D patients (*p* = 0.002672).

### 3.1. Subgroup Analysis for Surgical Technique

#### 3.1.1. SG

There was a total of 306 patients included in the SG group, with 87 patients (28.4%) in the T2D cohort and 219 patients (71.6%) in the non-T2D cohort. The mean age of patients in the T2D cohort was 42.8 years, whereas in the non-T2D cohort it was 31.6 years. The initial median BMI for the T2D cohort was 42.7, while for the non-T2D cohort it was 43.6 kg/m^2^. The gender distribution in the T2D cohort was 39.1% males and 60.9% females, while in the non-T2D cohort, it was 44.7% males and 55.3% females. The initial median HbA1c for the T2D cohort was 6.6, while for the non-T2D cohort it was 5.4. Importantly, both cohorts exhibited similar comorbidity profiles.

No statistically significant differences were observed in intraoperative findings. The average duration of surgery was 96.4 min in the T2D cohort and 89.2 min in the non-T2D cohort, showing a non-significant trend (*p* = 0.059198). The mean length of hospital stay did differ significantly, with T2D patients staying for an average of 3.1 days compared to 2.5 days for non-T2D patients (*p* = 0.00035). Early minor complications were observed in 8.0% of T2D patients and 8.6% of non-T2D patients, with no significant difference between the groups (*p* = 0.85716). Similarly, early major complications occurred in 5.7% of T2D patients and 2.7% of non-T2D patients, demonstrating no significant difference in complication rates (*p* = 0.20408).

Readmission rates within the early postoperative period were 4.5% for T2D patients and 3.1% for non-T2D patients, without a statistically significant distinction (*p* = 0.55520). Furthermore, reoperation rates were 2.2% for T2D patients and 3.1% for non-T2D patients, with no significant difference observed (*p* = 0.67448). Interestingly, there was no statistically significant difference in the mean BMI at 12 months of follow-up between T2D and non-T2D cohorts, indicating similar weight loss outcomes (33.3 kg/m^2^ for T2D vs. 31.4 kg/m^2^ for non-T2D, *p* = 0.196834). However, a significant difference was found in the mean HbA1c levels at 12 months of follow-up, with T2D patients showing an average HbA1c of 5.6 compared to 5.2 in non-T2D patients (*p* = 0.00573).

#### 3.1.2. RYGB

There was a total of 236 patients included in the SG group, with 73 patients (30.9%) in the T2D cohort and 163 patients (69.1%) in the non-T2D cohort. The mean age of patients in the T2D cohort was 46.4 years, whereas in the non-T2D cohort it was 34.9 years. The initial median BMI for the T2D cohort was 40.5, while for the non-T2D cohort it was 42.4 kg/m^2^. The gender distribution in the T2D cohort was 35.6% males and 64.4% females, while in the non-T2D cohort it was 30.7% males and 69.3% females. The initial median HbA1c for the T2D cohort was 7.6, while for the non-T2D cohort it was 5.6. Importantly, both cohorts exhibited similar comorbidity profiles.

No statistically significant differences were observed in intraoperative findings. The average duration of surgery was 167.8 min in the T2D cohort and 152.9 min in the non-T2D cohort, showing a non-significant trend (*p* = 0.14386). The mean length of hospital stay did not differ significantly, with T2D patients staying for an average of 2.84 days compared to 2.83 days for non-T2D patients (*p* = 0.98400). Early minor complications were observed in 2.7% of T2D patients and 0.6% of non-T2D patients, with no significant difference between the groups (*p* = 0.17702). Similarly, early major complications occurred in 2.7% of T2D patients and 5.5% of non-T2D patients, demonstrating no significant difference in complication rates (*p* = 0.34722).

Readmission rates within the early postoperative period were 4.1% for T2D patients and 4.9% for non-T2D patients, without a statistically significant distinction (*p* = 0.78716). Furthermore, reoperation rates were 2.7% for T2D patients and 3.0% for non-T2D patients, with no significant difference observed (*p* = 0.88866). Interestingly, there was no statistically significant difference in the mean BMI at 12 months of follow-up between T2D and non-T2D cohorts, indicating similar weight loss outcomes (30.4 kg/m^2^ for T2D vs. 31.6 kg/m^2^ for non-T2D, *p* = 0.35952). However, a significant difference was not found in the mean HbA1c levels at 12 months of follow-up, with T2D patients showing an average HbA1c of 6.0 compared to 5.4 in non-T2D patients (*p* = 0.216071).

## 4. Discussion

The main objective of this study was to compare the safety and postoperative outcomes between patients with and without T2D undergoing primary bariatric surgery at a tertiary referral academic medical center in the United Arab Emirates. Our findings revealed no statistically significant differences in any of these outcome categories between patients with T2D and those without T2D who underwent primary metabolic surgery.

Our study aimed to highlight the efficacy of these procedures for managing T2D in the MENA population, while also emphasizing their safety. Our results demonstrated that patients with T2D experienced a significant reduction in mean BMI by 11.6 points and an average decrease in HbA1c levels of 1.5%, from 7.2% to 5.7%. This decline in HbA1c was statistically significant and fell below the diagnostic criteria for T2D set by the American Diabetes Association. In this cohort, no significant differences in early minor (*p* = 0.67448) and early major (*p* = 0.81034) postoperative complications were found between T2D and non-T2D patients. Overall, when comparing our study’s results to previous international reference studies, we found our data to align with the reported safety profiles and rates of T2D resolution [14,15].

In light of the well-established safety and effectiveness of metabolic surgery in various populations worldwide [15,16], demonstrating long-term positive outcomes for individuals with and without T2D, it becomes crucial to examine its efficacy and safety in the MENA region. This region experiences a higher incidence rate of T2D, 19.5% compared to 10.5% in the United States, highlighting the need to validate the effectiveness and safety of metabolic surgery in this specific context. By conducting further research in the MENA region, we can enhance our understanding and provide valuable insights into the outcomes of this surgical approach [17].

Previous global studies have shown that patients with T2D are at a higher risk of experiencing postoperative complications, including but not limited to delayed extubation, circulatory dysfunction, diabetic acidosis, surgical site infections, pulmonary infections, respiratory failure, acute myocardial infarction (MI), and acute kidney failure. These adverse events have been proposed to be primarily influenced by poor preoperative glycemic management, which is considered a key risk factor [7].

In 2015, the University of Washington conducted a statewide cohort study that further supported this hypothesis. The study compared perioperative outcomes between hyperglycemic patients diagnosed with T2D and those without a T2D diagnosis undergoing both general and orthopedic surgery. The study included over 40,000 patients, out of which 3383 were found to have a perioperative blood glucose level higher than 180 mg/dL. Among these patients, a significant increase in the risk of adverse events was observed. Interestingly, there was also an elevated risk in the non-T2D group with hyperglycemia compared to the T2D group with hyperglycemia. This correlation was attributed to the non-T2D group’s lack of insulin use during the perioperative period [12,13].

In 2017, Raj et al. conducted a prospective study to evaluate the effectiveness of metabolic surgery as a treatment for Indian patients with T2D. Their study involved 53 obese patients with T2D, and the results showed an average reduction of 2% in HbA1c levels. Furthermore, an impressive 81.1% of the participants achieved T2D remission. However, the study did not include a discussion of complication rates [18].

Similarly, in 2015, Lee et al. published a prospective study documenting their experience with metabolic surgery as a treatment for Taiwanese patients with T2D. The study included 80 non-obese patients with T2D. The findings revealed an average decrease in BMI of 4.2 kg/m^2^ and a 25.0% incidence of complete T2D remission, with an additional 23.8% of patients experiencing partial remission. Among the participants, 11 patients experienced postoperative complications, with 2 cases classified as major complications (leakage and bleeding) and 9 cases as minor complications [19].

A systematic review and meta-analysis conducted by Soochow University encompassed data from 611 obese Chinese patients with T2D who underwent primary metabolic surgery. Their findings aligned with the aforementioned studies, indicating a decrease in total body weight, BMI, HbA1c, waist circumference, blood pressure, and fasting plasma glucose, all of which are indicators of T2D remission. The study also reported a mortality rate of 0.3% among obese Chinese patients undergoing primary metabolic surgery and a 15.9% complication rate. Among the complications, 13 were categorized as major complications, comprising 2.4% of the study population. The most commonly reported complications were bleeding, leakage, and stenosis of the anastomosis [20].

To our knowledge, no large-scale investigations conducted in the Middle East have been similar to our single-center study, despite the increasing popularity of metabolic surgery in the region, with 800,000 procedures performed between 2014 and 2019 [21]. This is particularly significant considering that the incidence of diabetes among individuals native to the MENA region is nearly double that of the United States (19.3% compared to 10.5%, respectively) [22].

## 5. Conclusions

In this cohort of patients from the MENA region, bariatric surgery in T2D patients is safe and effective, with perioperative outcomes comparable to those of non-T2D patients. To the best of our knowledge, our postoperative findings, which are the first report in the MENA region, are consistent with studies published in North America and Europe.

## Figures and Tables

**Table 1:** Baseline characteristics (N = 542).

Variables	T2D (N = 160)	Non-T2D (N = 382)	*p* Values
Age (Years, mean, SD)	44.5 (11.8)	33.1 (10.1)	0.00001
Male (N, %)	60 (37.5%)	148 (38.7%)	0.78716
Female (N, %)	100 (62.5%)	234 (61.3%)	0.78716
BMI (Median, kg/m^2^)	41.8	43.2	0.03824
Baseline HbA1c (Mean, SD)	7.2 (1.7)	5.4 (0.4)	0.00001
Triglycerides (Mean, SD)	1.8 (0.9)	1.8 (5.3)	0.92093
Duration of T2D (Years, mean, SD)	4.4 (6.9)	N/A	N/A
Medications for T2D (N, mean, SD)	1.0 (0.3)	N/A	N/A
Comorbidities			
Hypertension (N, %)	95 (59.3%)	91 (23.8%)	0.00001
Dyslipidemia (N, %)	114 (71.2%)	136 (35.6%)	0.00001
GERD (N, %)	48 (30.0%)	89 (23.2%)	0.10100
Obstructive Sleep Apnea (N, %)	63 (39.3%)	119 (31.1%)	0.06432
Coronary Artery Disease (N, %)	14 (8.7%)	8 (2.0%)	0.00034
Renal Insufficiency (N, %)	20 (12.5%)	7 (1.8%)	0.00001

Abbreviations: BMI, body mass index; HbA1c, hemoglobin A1c; GERD, gastroesophageal reflux disease; N/A, not applicable; SD, standard deviation; T2D, type 2 diabetes.

**Table 2:** Intraoperative findings (N = 542).

Variables	T2D (N = 160)	Non-T2D (N = 382)	*p* Values
Surgical Technique (N, %)			
RYGB	73 (45.7%)	163 (42.7%)	0.52870
Sleeve Gastrectomy	87 (54.3%)	219 (57.3%)	0.52870
Findings			
IOCs (N, %)	2 (1.2%)	2 (0.5%)	0.36812
Est. Procedure Length (min) (mean, SD)	117 (52.1)	114 (47.9)	0.057218

Abbreviations: RYGB, Roux-En-Y gastric bypass; IOC, intraoperative complication; SD, standard deviation.

**Table 3:** Comparison of peri- and postoperative outcomes (N = 542).

Variables	T2D (N = 160)	Non-T2D (N = 382)	*p* Values
Perioperative Findings			
Length of stay (median, days)	3	2	0.052873
Early minor complications (N, %)	7 (4.3%)	20 (5.2%)	0.67448
Early major complications (N, %)	7 (4.3%)	15 (3.9%)	0.81034
Readmission (N, %)	9 (5.6%)	19 (4.9%)	0.75656
Reoperations (N, %)	6 (3.7%)	6 (1.5%)	0.11642
Follow-Up			
BMI (mean, kg/m^2^, SD, at 12 months follow-up)	30.2 (6.1)	29.1 (7.2)	0.777767
HbA1c (mean, SD at 12 months follow-up)	5.7 (0.7)	5.2 (0.3)	0.002672
Triglycerides (mean, SD at 12 months follow-up)	1.2 (0.8)	0.9 (0.3)	0.02497
Follow-up (median, months)	14	10	0.000464
Mortality (N, %)	0 (0.0%)	0 (0.0%)	-

Abbreviations: BMI, body mass index; HbA1c, hemoglobin A1c; SD, standard deviation.

## Data Availability

Not applicable.
